# New Appliances and Things Medical

**Published:** 1896-05-30

**Authors:** 


### NEW APPLIANCES AND THINGS MEDICAL.

**[We shall be glad to receive, at our Office, 428, Strand, London, W.C., from the manufacturers, specimens of all new preparations and appliances, which may be brought out from time to time.]**

### LITMUS PENCIL.

(Thos. Christy and Co., 25, Lime Street, London, E.C.)

This is a most useful invention, and one that is quite certain to appeal to the busy practitioner. It is constructed in the form of an ordinary pencil, in which the lead core is replaced by a core of litmus—one end blue, the other end red. All that is necessary for testing the reaction of a specimen is to make a blue and a red mark on a piece of paper and dip t in the fluid; if the specimen is acid the blue mark turns red, if alkaline the red mark blue, if neutral there is no change in either. The litmus of which the core is composed is stated to be chemically pure, and to be capable of affording evidence of acid or alkali of one part in 100,000, comparing with one in 1,400 when ordinary litmus paper is employed. We cannot help thinking that the range of utility of this little invention might be still further extended if the pencils were made half their present size, so that they might be conveniently carried in the waistcoat pocket. The price is 1s. 3d. per pencil.

